# Help-seeking challenges for female sexual concerns: a qualitative study from Iran

**DOI:** 10.1186/s12913-023-09719-7

**Published:** 2023-06-29

**Authors:** Raziyeh Maasoumi, Shadi Sabetghadam, Afsaneh Keramat, Seyed Shahrokh Aghayan

**Affiliations:** 1 Department of Reproductive Health, School of Nursing and Midwifery, Tehran University of Medical Sciences, Tehran, Iran; 2 Nursing and Midwifery Care Research Center, School of Nursing and Midwifery, Tehran University of Medical Sciences, Tehran, Iran; 3 Student Research Committee, School of Nursing and Midwifery, University of Medical Sciences, Shahroud, Shahroud, Iran; 4 Reproductive Health research center, Guilan University of Medical Sciences, Rasht, Iran; 5 Department of Reproductive health, School of Nursing and Midwifery, Shahroud University of Medical Sciences, Shahroud, Iran; 6 Department of Clinical Sciences, School of Medicine, Shahroud University of Medical Sciences, Shahroud, Iran

**Keywords:** Sexual health, Sexual dysfunctions, Help-seeking behavior, Health services, Healthcare providers, Qualitative research

## Abstract

**Background:**

To effectively manage sexual health, people must have access to sexual health services. A small percentage of women with sexual concerns seek professional help. Hence, the need to explain the help-seeking challenges is felt from the perspective of women and health care providers.

**Methods:**

This study explored Iranian women’s challenges when seeking help for sexual concerns. Using a purposive sampling method, 26 in-depth interviews were conducted in Rasht in 2019–2020. The participants included sexually active women of reproductive age over 18 years old and 8 health care providers. The recorded interviews were transcribed and analyzed through content analysis.

**Results:**

From the description of 17 subthemes provided by the participants, two main themes were achieved, “Unfavorable sexuality development context” and “Inefficient sexual health services.”

**Conclusions:**

According to the results, it is suggested that policymakers pay more attention to the challenges women and health care providers face in help-seeking and promote sexuality education and sexual health services to achieve a higher level of help-seeking among women.

## Background

Access to services is the main concern in promoting sexual and reproductive health and rights [1]. Every day, people look for information and help for their sexual concerns, and some hope that the physician will ask them about such personal issues [2, 3]. Sexual health is a topic less controlled by physicians in routine visits. A small percentage of women with sexual concerns specifically seek professional help [4, 5]. Most healthcare professionals do not actively talk about sexual concerns with service recipients, showing the need for more attention to this issue [6, 7].

To effectively manage sexual health, people must have access to sexual health services. These include access to family planning services, looking for screening and treatment for sexually transmitted diseases, pre/post-abortion counseling, and support, growing concerns about sexual abuse and exploitation, and sexual health services to choose appropriate strategies to prevent sexually transmitted diseases and unwanted pregnancies. This helps them talk more easily about their sexual affairs and express their needs better [8]. In this way, they can adopt preventive behaviors, raise their awareness in sexual matters, share this information with others, gain a better understanding and accept the changes in their sexuality that occur over time, adopt healthy sexual behaviors, and consider categories such as pleasure and sexual violence and be more satisfied with the services provided [9].

Studies have shown that sexual concerns may negatively affect interpersonal relationships, quality of life, and intimacy in sexual relationships. Paying attention to sexual health is essential for maintaining psychological life satisfaction and quality of life [10–12]. Due to some cultural constraints and challenges in our community, in most cases, couples not only do not refer to specialists and counseling centers to solve sexual disorders but also refuse to raise this issue among themselves. This refusal can sometimes be the source of severe family disputes and, finally, emotional divorce [13].

The high burden of sexually transmitted diseases in young people shows they need more sexual health services. However, despite this issue, this age group has the most obstacles to accessing sexual health services. Some of these obstacles include concerns about confidentiality, anonymity, being stigmatized, shame and embarrassment, fear of examination, and unknowing of what services are provided or how to access them [14].

A qualitative study in Turkey showed two factors affecting help-seeking for mental health issues, social stigma and reluctance to share problems with a stranger [15]. Another study in Norway showed that factors affecting seeking help for sexual concerns in older people are dynamics in communication, understanding of sexual desires, knowledge and competence, attitudes, and structural conditions [16]. However, Iranian culture is conservative in sexual matters, which affects their sexual health behaviors [17, 18]. Considering the role of help-seeking in improving women’s sexual quality of life, and since most related studies in our community are quantitative [4, 19–21], a qualitative study of this phenomenon can be useful to explain help-seeking challenges felt by women and health care providers’ viewpoints. Therefore, this research was conducted to explain the challenges of women’ help-seeking for sexual concerns.

## Methods

This study was conducted in Rasht, northern Iran, between December 2019 and August 2020. It is a qualitative study that uses the conventional content analysis technique. The investigation was conducted at three gynecological and psychology clinics in Rasht, in addition to two chosen healthcare facilities located throughout the city. There were 26 people in the research population (18 women and 8 health care providers). Purposive sampling was carried out with maximal diversity until saturation was attained. ShS, the corresponding author, used semi-structured and in-depth interviews for data collection.

Several women’s cellphone numbers were first obtained from the Integrated Health System with the consent of the health facilities. After being contacted and informed of the research objectives, some decided to participate in the interviews. As service receivers, they were then informed of the date, time, and location of the interviews.

In the section on the collection of data relating to health care providers, healthcare professionals who had knowledge and expertise in sexual health services and were willing to take part in the study were enlisted. With previous arrangements, their informed consent, and the protection of their privacy, they were interviewed at their place of service or in private clinics. Initially, 18 semi-structured interviews with women were conducted, of which 13 involved women with academic degrees and the remaining five involved women without academic degrees, keeping in mind the widest possible age variation (Table 1). Moreover, 8 key informant interviews were conducted with health care providers, including 1 psychiatrist, 1 clinical psychologist, 1 gynecologist, 3 midwives, and 3 reproductive health experts (Table 2).

The selection criteria for the women of reproductive age included being at least 18 years old, having Iranian nationality, being literate, engaging in sexual activity, not being pregnant, not being in the puerperium, not working in healthcare, and being willing to join the study. The health care providers were chosen from candidates with at least three years of professional expertise in providing sexual health services. Every possible consideration was taken into account to ensure that the health care providers include a wide range of ages, work experience, and areas of expertise.

**Table 1:** Details of women

No.	Age	Spouse age	Education	Spouse Education	Job	Spouse job	Marriage duration
1	23	30	High school diploma	High school diploma	Housewife	Self- employed	4
2	30	32	High school diploma	Bachelor’s degree	Housewife	Military	8
3	26	40	High school diploma	Bachelor’s degree	Housewife	Self- employed	2 (Second marriage)
4	21	29	Bachelor’s degree student	Bachelor’s degree	Student	Self- employed	1
5	32	32	PhD	Master’s degree	Employee	Self- employed	5
6	25	28	Bachelor’s degree	Bachelor’s degree	Employee	Employee	5 (Second marriage)
7	32	35	Master’s degree	Bachelor’s degree	Employee	Self- employed	8
8	29	33	High school diploma	Under High school diploma	Housewife	Driver	10
9	35	31	Master’s degree	Master’s degree	Employee	Shopkeeper	5
10	32	32	Bachelor’s degree	High school diploma	Kindergarten teacher	Carpenter	4
11	27	27	Bachelor’s degree	Associate degree	Employee	Military	2
12	37	25	Master’s degree	Bachelor’s degree	Self- employed	Self- employed	3 (Sexual relations without marriage)
13	24	27	Master’s degree student	Master’s degree	Employee	Employee	2
14	33	33	Master’s degree	Bachelor’s degree	Employee	Employee	15
15	38	39	Master’s degree	Bachelor’s degree	Employee	Employee	3
16	30	32	Bachelor’s degree	Bachelor’s degree	Self- employed	Self- employed	6
17	39	40	Bachelor’s degree	Master’s degree	Employee	Employee	12
18	24	25	Under High school diploma	High school diploma	Housewife	Self- employed	1 (second marriage)

**Table 2:** Details of Healthcare providers

	Age	Specialty	Workplace	Professional experience (Years)
1	32	Midwifery (Master’s degree)	Private clinic	5
2	40	Reproductive health	Private clinic	12
3	38	Reproductive health	Private clinic	10
4	45	Midwifery (Bachelor’s degree)	Health center	15
5	44	Reproductive health	Private clinic	4
6	37	Clinical Psychologist (Master’s degree)	Private clinic	8
7	43	Psychiatrist	Private clinic	10
8	36	Gynecologist	Private clinic and hospital	5

The interviews were semi-structured, and ShS began the data collection in the women’s section with a broad and open inquiry: “Have you ever encountered a sexual concern or problem?” If so, what did you do? “Why do not you see a sexual therapist for sexual concerns?”, “What steps have you taken to address your sexual concerns?” “Have you ever had the experience of seeing a sex therapist?” and further proceeded by asking exploratory questions like, “Has attending the center and obtaining services been helpful?” if yes, “Which of the characteristic of the service you received met your need?“ if not, how should your required service be?” in health care provider section ShS investigated by asking questions like “What do you do for your client’s sexual concerns?” and “What obstacles do you see for women to seek help for sexual concerns?”, “Have the services provided by you been able to give your clients sexual health?” If so, what characteristics did your services possess? If not, what characteristics should these services have? The time and place of the interviews were selected according to the participants’’ choices and conditions. For women, most interviews were conducted in a private room at a health center or in public spaces such as parks or coffee shops. For health care providers, interviews were conducted at their offices. The interviews lasted 30–90 min. When we did not get any new code in the last interview, we conducted two more interviews to avoid false theoretical saturation. Considering that we did not find any new codes, we ensured that theoretical saturation was achieved.

### Data analysis

Conventional content analysis was employed to analyze the data (Granheim and Lundman, 2004). Based on this pattern, the corresponding author (ShS) immediately recorded and transcribed each interview at the first possible opportunity. The semantic units, a group of words, each with a distinct meaning or notion related to the research objective, were summarized, and relevant primary codes were grouped into one subcategory. Subcategories were then evaluated repeatedly and, based on their differences and similarities, were assigned to a category. Similar categories made the main category [22]. An example of the coding process is presented in Table 3. Using MAXQDA 2010, the textual data and extracted codes were managed.

### Trustworthiness

We employed the approach suggested by Lincoln and Gabba to assess and improve the scientific rigor of the findings. The prolonged engagement with the data, data immersion, efficiency of in-depth interviews in multiple sessions, and member checking all contributed to the data’s credibility. The interviews were integrated into a Word file to facilitate member checking. To evaluate the precision of the transcriptions, three of these files were presented to three participants. External check and peer debriefing were conducted by the research group and two faculty members specializing in qualitative research to confirm the validity of the recorded interviews and the extracted codes and categories.

Three experts in reproductive and sexual health who were not members of the study team, but were knowledgeable in qualitative research for supervision of the data analysis and the use of their confirmatory or critical feedback, evaluated the data reliability. Confirmability was achieved by precisely registering and documenting the path of the investigation and the decisions taken. The women were chosen with the most age and socioeconomic diversity in mind for transferability concerns [23].

**Table 3:** An example of code analysis

Category	Subcategories	Codes	Quotes
Unfavorable sexuality development context	Weakness in sexuality knowledge	Lack of sexual body cognition	No one has any information about their own body. What if they want to have sex with another person and reach a sexual agreement? Many people may not understand what these problems are related to
	Lack of information about sexual rights	Lack of awareness of the right to protest against sexual harassment	We also lack awareness [about sexual rights]. For example, I had a case that her husband wanted her to play a role like her sister during sex. She said that this is annoying for her, but she does not know that she has the right to protest and say no to this sexual request

## Findings

The women’s age ranged from 21 to 39 years, and their mean age was 29.8 years. The health care providers’ age ranged from 32 to 45 years, and their mean age was 39.4 years. Further details of the participants’’ demographic characteristics (health care providers and women) were presented in Tables 1 and 2.

**Fig. 1:**
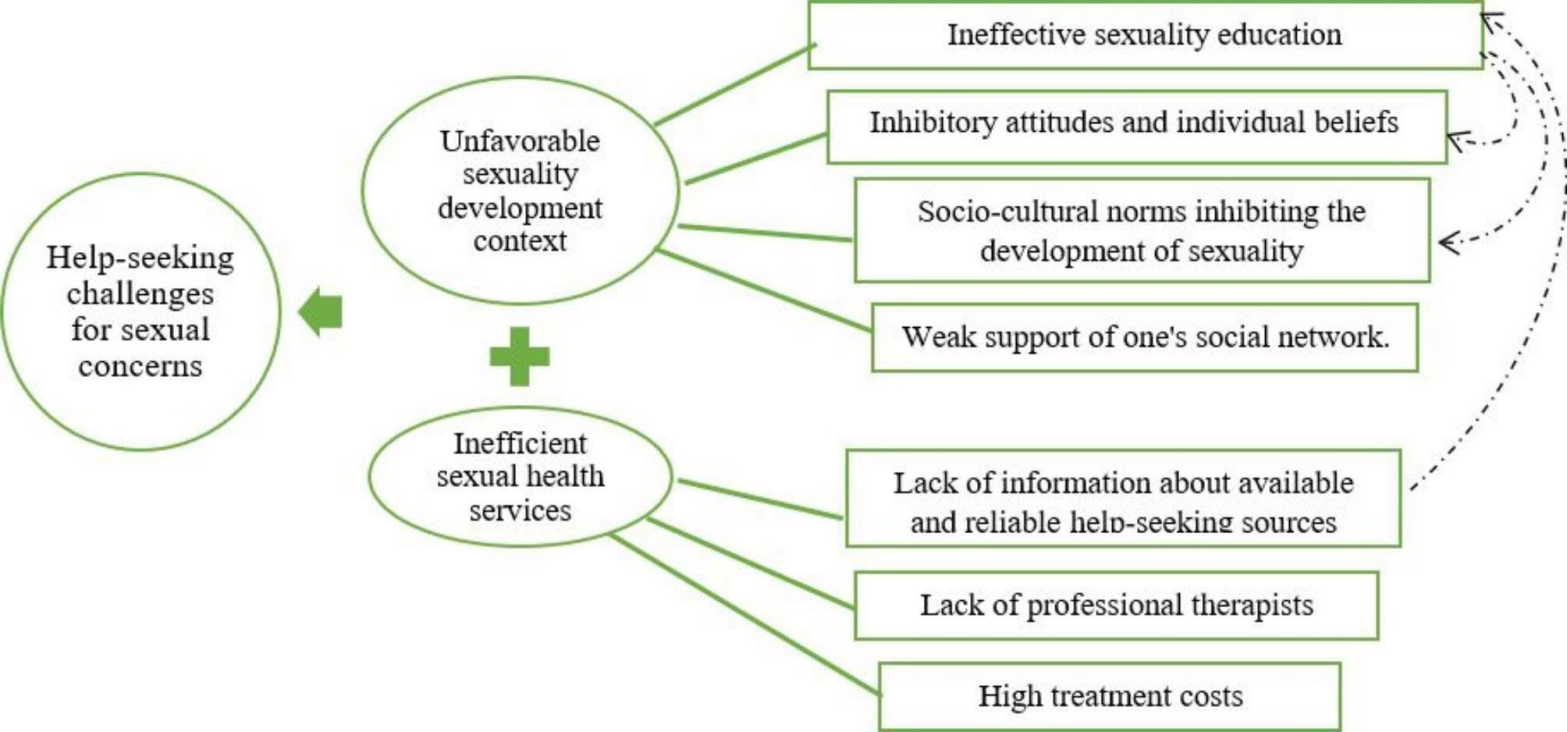
Overall schema of challenges in seeking help for women’s sexual concerns

Data analysis resulted in two main categories, “unfavorable sexuality development context” and “Inefficient sexual health services.” “Unfavorable sexuality development context” consists of four categories, including “ineffective sexuality education,” “sociocultural norms inhibiting the development of sexuality,” “inhibitory attitudes and individual beliefs,” and “weak support of one’s social network,” and 11 subcategories. “Inefficient sexual health services” consists of three categories, including “lack of access to reliable help-seeking resources,” “lack of professional therapists,” “high treatment costs,” and six subcategories. The categories, subcategories and codes were presented in Table 4, and the overall schema of challenges in seeking help for sexual concerns is illustrated in Fig. 1.

**Table 4:** The main categories and subcategories of help-seeking challenges for sexual concerns among women

Main Category	Categories	Subcategories	Codes
Unfavorable sexuality development context	Ineffective sexuality education	Weakness in sexuality knowledge	Lack of enough knowledge about sexual issues, Lack of sufficient information in premarital education, lack of sexual body cognition, low sexual experience.
		Lack of information about sexual rights	Ignoring sexual rights, sexual compliance roles, lack of awareness of the right to protest against sexual harassment.
		Improper sexuality education for children	Lack of sexuality education in the family, failure to teach the importance of sexual issues in school, the necessity of teaching self-esteem to teenagers.
	Inhibitory attitudes and individual beliefs	Prioritizing other life issues and ignoring sexual matters	Problems and preoccupations in life prevent help-seeking, not paying attention to sexual concerns, failure to visit due to husband’s busyness.
		Misconceptions about treatment	Belief in self-treatment, belief in the ineffectiveness of treatments, fear of facing a possible problem. If I get help for my husband’s problem, he will be disappointed, and I may have surgery. Female orgasmic dysfunction is normal, low desire in women is normal, some races are not sexually hot.
		Not valuing for herself	Prioritizing the satisfaction of the spouse, lack of self-love in women. the need to strengthen women’s self-esteem.
	Sociocultural norms inhibiting the development of sexuality	Sexual concerns as taboo	The need to put aside ugly sexual issues, the difficulty of talking about sex, a conservative community about sexuality, fear of judgment from spouse and others, embarrassment of gynecology examination.
		The impact of religious teaching on sexuality	Considering sexual affairs as ugly due to religious teachings, Feeling guilty about sex due to religious teachings, and considering sexual concerns as ugly due to the traditional primary family.
		Patriarchal Community	The atmosphere of the community favors men, suppressing women’s issues in our culture and considering women as the second gender in the community.
	Weak support of one’s social network	Undesirable interpersonal relationship with the spouse	The need to talk with the husband about sexual concerns, lack of interest in the couple, It is considered ugly to talk about sexual issues with the husband.
		Lack of support from spouse and family	The necessity of accompanying the spouse to visit a physician, lack of support from the primary family, the need for a trusted person to accompany, people around you as a model of help-seeking behavior.
Inefficient sexual health services	Lack of access to reliable help-seeking resources	Lack of information about available and reliable help-seeking sources	Lack of information on where to go for sexual concerns, the necessity of informing about sources of help, the need for a reliable source, lack of suitable educational resources, using porn for education, non-educational information on the internet, lack of coordination of some educations with religious beliefs.
		Lack of access to a skilled therapist	Absence of centers for sexual health services, lack of experts in the field of sexual health, the necessity of having the possibility of non-attendance consultation, lack of access to a female specialist, the necessity of screening for sexual concerns, healthcare worker time limit for screening in health centers, the reluctance of some people to go to government centers.
	Lack of professional therapists	Therapist’s undesirable communication skills	Favorable behavior of the therapist, negative judgment by the therapist, a physician should ask first, lack of privacy in the physician’s office, lack of confidentiality, not spending enough time by a therapist, the lack of importance of sexual health for service providers, a therapist should speak in plain language, the client’s experience in the first treatment session should be satisfactory.
		Therapist’s undesirable specialized skills	Need to have the necessary knowledge for sexual therapy, incorrect recommendations of a therapist, prescribing only drugs for sexual concerns, incompatibility of therapists’ knowledge with religion, failure to refer to other specialists if necessary, the necessity of investigating the problem by a treatment team, the need for the therapist to be experienced, the need for considering the difference between people.
	High treatment costs	Financial inability	Financial problems, the high cost of counseling, the need for free or low-cost sexual health services, people not valuing free services.
		Lack of health insurance coverage	The necessity of insurance coverage for sexual concerns, treatment-oriented insurance.

### Unfavorable sexuality development context

This main category describes the most important help-seeking challenge in the community, which is an unfavorable context for the proper development of sexuality. This unfavorable context includes the lack of effective sexuality education, inhibitory sexual attitudes and beliefs, and sociocultural barriers (the latter two are due to the lack of education), as well as the lack of a supportive social network, which are other obstacles to women’s help-seeking in our context. This main category included 11 subcategories, as explained below.

#### Weakness in sexuality knowledge

All women and health care providers believe that weakness in sexuality knowledge is an essential challenge for help-seeking about sexual concerns. Due to the lack of effective sexuality education, women have little sexual knowledge. A 32-year-old Ph.D. student said:

*“No one has any information about their own body. What if they want to have sex with another person and reach a sexual agreement? Many people may not understand what these problems are related to”* (Participant No. 5).

Even with premarital education courses, effective sexuality education does not happen. A reproductive health specialist with ten years of experience referred to sexual content provided to couples when they get married in Iran. She believed that premarital education courses are ineffective and said:

*“The premarital courses are not held with proper quality. I think it even has a bad effect. They usually teach the belief, based on which sex is a woman’s duty, and this belief is reinforced that a woman has no right in sexual matters”* (Health care provider No. 3).

#### Lack of information about sexual rights

Some women and health care providers referred to this issue that women do not know about sexual rights, which prevents them from seeking help. In this regard, two healthcare providers said:

“*We also lack awareness* [about sexual rights]. *For example, I had a case that her husband wanted her to play a role like her sister during sex. She said this is annoying, but she does not know she has the right to protest and say no to this sexual request”* (Health care provider No. 6).

*“Regarding sexual matters, the view of having sex as a commodity and service by women exists because of the community’s laws. According to the law, a woman must obey. The man should give alimony to her, and the woman must obey. While we now know that sex is not something in life, so the husband pays the living expenses, and the woman has sex with him in return. This should be amended in the laws”* (Health care provider No. 7).

#### Improper sex education for children

Some women and health care providers believe that the lack of sexuality education for children makes them unprepared to face sexual concerns and seek help in adulthood. A 27-year-old woman said:

*“I think it is important that families do not say a series of words to their children that will cause fear and shame about sexual concerns in the future. They should not be so sensitive to the opinions of others. For example, “Wow, what will people say if you do this?” I think that this shame created in mind from childhood makes a person unable to talk about his/her sexual concerns”* (Participant No. 11).

#### Prioritizing other life issues and ignoring sexual matters

For some women, sex was not an important issue. They believed that despite the more important problems in life, why they should care about sexual concerns. Some participants were satisfied with the priority of their husband’s satisfaction Some participants raised the importance of other issues in life. A 30-year-old woman said:

*“I had so many difficult problems in my life, so I do not see any sexual concerns anymore. I have had so many career, financial, and family problems that sexual concerns are no longer important to me*” (Participant No. 2).

#### Misconceptions about treatment

In our context, misconceptions about treatment were another challenge in help-seeking for sexual concerns. According to some participants with sexual concerns, it is a part of being a woman and normal. Some of them believed they could solve the problem by self-medication, or they thought there was no cure for their problem. One of the health care providers said in this regard:

*“Unfortunately, some people consider themselves know-it-alls. They think that they can find the solution by themselves. They search on the internet and watch porn. As a result, they satisfy themselves that they know what to do”* (Health care provider No. 4).

One woman said she feared facing the truth about her sexual concern. She was worried about whether her sexual fantasy was right or wrong and expressed her fear of help-seeking as follows.

*“Since I discovered that there is a field of psychology called sexology, I have wanted to get help on this issue, but I did not get help. To be honest, I was afraid that if I approached them, they would tell me that this [sexual fantasy] was wrong. It was something that I did not want. I was afraid of that negative answer. That is why I did not get help” (Participant No. 8).*

#### Not valuing herself

Another challenge some women and health care providers raised to seek help was that women do not value themselves or have enough self-esteem. This makes them not pay attention to their sexual concerns and not seek to solve their sexual concerns. A 21-year-old woman said:

*“When a woman does not love herself, she does not care about herself and tolerates problems, even sexual concerns, because she does not consider herself worthy enough to go and solve them”* (*Participant* No. 4).

#### Sexual concerns as taboo

Almost all the participants believed that the community is very conservative about sexual concerns, which is taboo in the culture and prevents raising sexual concerns with the health care provider. They believed there was a need to break the taboo in the community. A 26-year-old woman with two years of a second marriage and having difficulty in reaching orgasm said:

*“I was embarrassed to go to the physician. I tell someone I cannot reach orgasm!?”* (Participant No. 3).

#### The impact of religious teaching on sexuality

Although, in Islamic teachings, sex and sexuality are not considered sinful or ugly, some women’s perceptions of religious teachings have made them consider sexual concerns ugly or sinful, affecting help-seeking for sexual concerns. A 32 years old woman, Ph.D., married for five years, said:

*“I think one of the reasons* [for not seeking help] *is that our culture is religious, and our religious teachings have made people consider sex ugly. In other countries, children can learn about sexual concerns from a very young age and even experience sex”* (Participant No. 5).

#### Patriarchal community

A few participants referred to the patriarchal community as a challenge for women to seek help for sexual concerns. They mentioned that the atmosphere of the community is in favor of men. Women are the second gender, and issues related to them are suppressed. One of the health care providers stated that because of the patriarchal community, women do not come with their chief complaint, but even when they go to the service provider, they bring up their husband’s problem. A woman said:

*“Women are still considered the second gender. When a woman discusses sexual concerns with the physician, even the physicians may have something like this in their mind”* (Participant No. 7).

#### Undesirable interpersonal relationship with a spouse

In this regard, challenges, including unfavorable interpersonal relationships with the spouse, lack of support from the spouse, and lack of support from family and relatives, were raised. A 30-year-old woman who did not have an excellent interpersonal relationship with her husband said:

*“My husband annoys me so much that I don’t care if I’m satisfied with sex anymore. If I knew that my husband wanted me wholeheartedly, it would make me want to solve my sexual concerns”* (Participant No. 2).

#### Lack of support from spouse and family

For a few women, not having a trusted family member to seek medical help for sexual concerns was a challenge. A woman referred to the need for her husband’s support to accompany her to get help.

*“Well, the best thing is the husband’s support. The husband should support and accompany her. He should accompany the man if he agrees to come. Only if he comes, says something, who heard that he accepted the problem. Well, my husband was unsatisfied to come”* (Participant No. 10).

### Inefficient sexual health services

The other important challenge in help-seeking for sexual concerns was inefficient sexual health services. Both women and health care providers had thoughts about how sexual health services might influence help-seeking. This main category included six subcategories, as explained below.

#### Lack of information about available and reliable help-seeking sources

Some women stated that there is no reliable official (sexologists and other health care providers) and unofficial (books, websites, etc.) sources to get information and solve sexual concerns. A 21-year-old woman said:

*“Information is not enough. It is not given any importance in the media. For example, as soon as you called to do your thesis, how excellent it would be if the health center would be informed about everyone’s* [sexual health] *conditions by a call, or at least informed them that you could go to your local health center. Let me also say that I was happy when I heard your voice saying that you are from the health center because I have a series of unresolved sexual concerns that I do not know how to solve”* (Participant No. 4).

Some health care providers stated that there are not enough useful sexuality education sources and content so that women could obtain sexual information and be guided towards recognizing their sexual concerns and seeking help. A midwife believed that:

*There is not enough information about sexuality and sexual help sourcesMany of the Internet contents are incorrect and even cause a series of false sexual beliefs to be formed”* (Health care provider No. 1).

#### Lack of access to a skilled therapist

Some women and health care providers believed there were not enough skilled therapists available. They believed that there was no specialist in the field of sexual concerns whose skills they could trust. Some pointed out that there is no female specialist, and one of the participants thought he should go to the capital to find a skilled specialist. She said:

*“If you want to visit a good physician, you must overcome many difficulties. These services [sexual health services] should be more available. I should not have to take time off from work, go to another city and spend much money to solve my problem”* (Participant No. 6).

#### Therapist’s undesirable communication skills

Some participants referred to the impact of therapists’ unfavorable communication on help-seeking. They complained that many health care providers do not spend enough time, do not ask questions about sexual concerns, do not treat clients politely, are judgmental, and cannot be trusted easily. A woman referred to the importance of not being judged by the therapist and said:

*“The counselor’s open and enlightened vision for talking without considering the marital status makes one more willing to go to the counselor. But unfortunately, there are very few people with these characteristics. If I discuss my sexual problem with someone, it should be a person with an open mind, without judgment and bias”* (Participant No. 4).

#### Therapist’s undesirable specialized skills

In the present study, most participants referred to the challenge of the lack of therapists’ specialized skills. Some women believed that service providers often do not have the necessary skills to manage sexual concerns. Some service providers believe that treating sexual concerns should be done as a team, but currently, most psychiatrists only prescribe drugs, and psychologists are ignorant of sexual anatomy and physiology. Midwives and gynecologists who deal with women a lot in Iran do not have enough knowledge and skills. In this regard, a young gynecologist pointed out the lack of skills of gynecology residents for sexual concerns and said:

*“We do not have sexology in our curriculum. This is a prevalent complaint among women, and we have no training for it. To be honest, the first patient that came in, I realized that I knew nothing about treating sexual concerns. For example, I know that certain drugs reduce libido because I worked as a physician*” (Health care provider No. 8).

#### Financial inability

Some women stated they could not go to service providers for their sexual concerns due to financial problems, and some referred to the high costs of counseling sessions. Some service providers pointed out that it is necessary to reduce treatment costs. However, at the same time, free sexual health services should not be provided because clients usually consider free services worthless. A woman said:

*“I think that the main problem of our community is the financial problem. If there is no financial problem, one can easily go to different physicians and get check-ups. She can go to any physician as often as she wants*” (Participant No. 10).

#### Lack of health insurance coverage

A few participants (both women and health care providers) stated that counseling for sexual concerns is not covered by health insurance. They considered this a challenge to get help from formal help-seeking sources. One health care provider raised health insurance issues with sex counseling services and said:

*Insurance in our country is generally treatment-oriented. Insurance is willing to pay 700 million IRR for a patient hospitalized in ICU, but it does not pay 20 million IRR for ten counseling sessions of sex therapy. Insurance companies do not pay for counseling”* (Health care provider No. 5).

## Discussion

The views and experiences of the participants to answer the research question “How do women explain their challenges in seeking help for sexual concerns?” were presented in the form of two themes: “unfavorable sexuality development context” and “inefficient sexual health services.” This study highlighted the challenges women face in seeking help for sexual concerns. We showed that women in our community lack enough knowledge about sexual health and rights. As a result of this limited knowledge, there are wrong beliefs about sex and seeking help rooted in a sexually conservative Community. Even when deciding to seek help, not being accompanied by a spouse or a trusted person is another challenge for women to seek help for sexual concerns.

We showed that inefficient sexual health services in our community are another challenge for women to seek help. Even if women decide to seek help for sexual concerns, they face challenges due to a lack of information about available and reliable health care providers. Even if they find a skilled specialist, the high costs of counseling and treatment prevent them from therapy.

Our study highlighted the profound impact of the lack of effective sexuality education and its challenges to help-seeking. Few previous qualitative studies on help-seeking for women’s sexual concerns have addressed this issue. All participants believed that ineffective sexuality education is the main challenge for women in help-seeking for sexual concerns. The lack of proper sexuality education makes it challenging to recognize sexual concerns and the need to seek help, women are unaware of their sexual rights, and they do not know reliable help sources. A recent study in Iran showed that a quarter of women in Tehran had limited sexual health literacy [24]. Sexual health literacy increases the ability to understand and evaluate sexual health risks and correctly understand one’s duties and responsibilities in sexual relations in a person’s [25–27]. A qualitative study in Norway showed that the lack of sexual awareness due to cultural factors and rural life in the past and not learning about sexual concerns at home and school had been one of the obstacles to seeking help for older people [16].

According to the woman and the health care providers, inhibiting sociocultural norms was another challenge for women to seek help in sexual matters. Sexual matters are taboo in most communities. Self-stigma and embarrassment are the most critical barrier for people talking to a health care provider about sexual concerns [28, 29]. In Eastern cultures, especially Islamic cultures, sexual concerns are taboo, and people rarely discuss sexual concerns [30, 31]. The comparison of our findings with the previous findings shows a significant similarity. For example, Maasoumi et al. (2019) reported that help-seeking from religion through actions, including praying, pilgrimage, or making offerings, is one of the basic strategies that Iranian people, especially women, use in many problematic situations, including sexual concerns. However, there is a general difference of opinion about the effect of religion on women’s sexuality [17, 18, 32–34]. Maasoumi et al. described the need for sexual health services according to the sociocultural background of people [35].

We show that, besides sociocultural barriers, some attitudes and beliefs in women prevent help-seeking. Women have misconceptions about their self-worth, sexuality, and sexual therapies. The point of view of some women who did not seek help for sexual concerns was that they have more important problems in life, and therefore sexual concerns are unimportant. Moreira et al. (2008) found a significant relationship between the importance of sexual relations and formal help-seeking in women. In this study, women who cared about their sexual concerns were about 3.5 times more likely to seek medical help for sexual concerns [36].

As a result of the patriarchal culture, some women did not attach importance to their sexual satisfaction and declared that only their husband’s sexual satisfaction was important. In line with this finding, a qualitative study in Malaysia showed that Malay women considered sex to mean sexual intimacy in marriage and preferred their marital role as a “good wife” over their rights in a sexual relationship [37].

Weak support of women’s social networks was the other challenge in help-seeking for sexual concerns. The lack of a favorable emotional relationship with the spouse and the absence of accompanying by the spouse, family, or relatives prevented some women from seeking help for sexual concerns. A study regarding students’ help-seeking showed that students who were aware of the help of others (e.g., family, friends) were twice as likely to seek formal (e.g., psychologist) and informal (e.g., cleric) help-seeking [38].

Many women and health care providers believe that lack of access to reliable help-seeking resources is an important challenge in help-seeking. Another qualitative study in Poland also confirmed the lack of information on how to access appropriate services [39]. This lack of sufficient formal and informal sources for help-seeking also leads to inadequate knowledge about sexual health and rights.

The lack of professional therapists was another important challenge. Some women and health care providers believe that there are few skilled professionals in sexual health. A mixed method study in postpartum women also showed that even when women sought help from health care providers, they were not getting a helpful response [40]. Some of our study participants believed that some health care providers do not have enough communication skills to communicate with clients and raise sexual concerns properly. The therapist’s favorable communication skills increase using services [41]. Other studies also showed that communication skills are effective in seeking help for sexual concerns [16, 39]. Most clients wanted the health care provider to ask about sexual health concerns [29]. A qualitative study on older people showed that the patient’s fear of not being taken seriously and the physician’s silence to ask caused the patient’s sexual concern not to be discussed [42].

Finally, some women and health care providers referred to the high costs of treating sexual concerns. Unfortunately, sexual counseling is not covered by insurance in our country, and considering that treating sexual concerns requires multiple visits, it is expensive for clients. Studies have shown that reducing the direct costs of care and insurance coverage can increase using sexual health services [43, 44].

This was the first study that explained the challenges of women’s help-seeking for sexual concerns in an Islamic community. The results highlighted the impact of the lack of effective sexuality education on women’s help-seeking. However, the study had some limitations, including the sensitivity of the sexual subject, which affected the participants’ incentives and inclination to enter the study. By describing the research objectives and building trust, the researcher attempted to manage this limitation. In this research, the help-seeking challenges of pregnant and postmenopausal women were not investigated due to their special conditions. It is suggested to clarify the understanding and experiences of these groups in help-seeking in future studies. Finally, we had a prolonged sampling duration due to the covid 19 pandemic limitations.

## Conclusion

This study showed women’s challenges in getting help for sexual concerns. Women do not have enough knowledge about sexual health and rights. Therefore, there are misconceptions about sexuality and help-seeking for it. Due to the sexually conservative community and sometimes the absence of a spouse or a trusted person, women do not seek help for sexual concerns. On the other hand, sexual health services are inefficient, women do not have enough information about available and reliable help-seeking sources, and the costs of treating sexual concerns are high. Hence, it is suggested that policymakers pay more attention to the challenges women face in seeking help and promote sexuality education and sexual health services to achieve a higher level of help-seeking among women.

## Data Availability

The data that support the findings of this study are available from the corresponding author [ShS] upon reasonable request.
